# Doctors of Osteopathy

**Published:** 1897-09

**Authors:** 


### DOCTORS OF OSTEOPATHY.

The latest and most absurd addition to medical sectarianism hails from Missouri. The founder is a former medical man, A. J. Still. He claims to have a wonderful discovery in the form of a "new science" of medicine. Many years ago, we are told, he had a daughter who was afflicted with spinal meningitis, and whom the most heroic efforts of both himself and other able physicians were unable to save. This set him to brooding over his loss, and out of this sorrow he finally evolved the theory that the proper treatment of disease is not one of drugs, but of manipulations of nerves, muscles, and bones. By a process of inner cogitation he perfected what he calls his "new science." For twenty years he struggled along in poverty, no one believing in him until about four years ago, when by a dexterous turn in advertising he found himself famous. Already he is said to be worth $200,000, and has more patients and students than he can attend to. The State of Missouri gave him a charter to establish a college where the degree of D. O. (Doctor of Osteopathy) is conferred. Other charters have been obtained from several States, and an organized effort has been made to get exemption for osteopathic students from the examinations of qualification other practitioners have to undergo. They seek such exemption on the plea that they administer no medicines and use no knives, therefore endanger no lives. Ingenious as this plea is, it did not work as well as they expected it to in Illinois. It passed current with the Legislature and was accepted as gospel by the State Senate, but the Governor, though expressing sympathy with their "science," vetoed their bill on the grounds of its unconstitutionality. Governor Tanner rightly held that it was a case of class legislation. In his veto message he said: "If by my veto I was subjecting the adherent of the new system to any undue hardships or depriving them of any rights enjoyed by others in the laudable business of healing the afflicted, I would hesitate, but I am not." If the osteopathy bill had passed unvetoed it would have destroyed the usefulness of the State Board of Health and demoralized medical practice in Illinois. The strength of osteopathy at present seems to exist in its political pull. A number of influential politicians have taken it up and are seeking to boom it. In Ohio they have a strong advocate in Senator Foraker, by whose personal influence the system was legalized in that State. The Senator claims that his son Arthur was cured of some form of chronic illness by osteopathic treatment. In an interview with Miss Ada M. Peck, D. O., of Toledo, Ohio, by a reporter of the Toledo *Blade*, she tells the world that "In the case of Arthur Foraker the cure was easy, as he was very young and his bones were soft. The manipulation in his case was the intercostal nerve through the ribs, and the pneumogastric nerve in the neck." The reporter asked Miss Peck to tell him what osteopathy is, and this is her reply: "Osteopathy is a science based upon anatomy and physiology. Armed with a comprehensive knowledge of the human body, the osteopath is enabled to trace disease to some abnormal position of bone, contraction of muscles, etc., obstructing the nerve-force, or the circulation of blood, or other vital forces of the body. By his thorough knowledge of the anatomical structure of the body the osteopath, by manipulation adjusts the abnormality, thus establishing nerve-force and circulation." While to an educated medical man this theory is simply ridiculous jargon, to the layman it passes current for wisdom. They have no means at their disposal to test its merits or pass upon its truth. For us to denounce such drivel is simply to make the average man of business think that we are envious or prejudiced. How, then, can we direct the minds of men so influential as Senator Foraker into the paths of medical common sense? It certainly is not a lack of ordinary intelligence that has carried him into such a camp. He is as shrewd a man, outside of medical matters, as there is in the United States. He and others like him should be taught that in the ranks of regular medical practice his son could have received the same treatment as was given by the osteopaths. He should be shown that Still and his followers do nothing that has not been done for centuries by regulars, masseurs, spiritualists, healers, bonesetters and others. He should be taught that the only new thing about the new (?) system he has espoused is its lies and dangers. Men like him should know enough about the doctrine of probabilities to perceive that the cure of his son by these people is no evidence of the universal merit of their system. Shyster lawyers do sometimes win the cases that they try while the most skillful lawyers sometimes fail. What would Senator Foraker think of a client, who, because a good lawyer lost a case for him and a bad lawyer on appeal won it, should ever after prefer the poor and disreputable lawyers to the good ones, and use his influence to get everybody to oppose the latter? Medicine is as uncertain in many directions as law, but wise men genererally prefer to trust themselves in the hands of those having the highest recommendations rather than in those of men with no reputation. In every department of life the men equipped with the most thorough education in their line, other things being equal, are the safest men to trust. It is a plain mark of eccentricity for anyone to keep continually choosing the irregular hangers-on in any line or profession. Senator Foraker's own strength is in his regularity and his honorable adherence to his party. If Still had stuck to his manipulations of bones, nerves and muscles within the ranks of the regulars no one would ever have interfered with him. He could even have made a specialty of his treatment and remained regular. He could not, however, make manifestly false claims therefor, nor have been able to advertise himself by crying out that the "allopaths" were persecuting him. The cry of "allopathic persecution" is most of the stock in trade of nearly every sectarian. They always take particular pains to hide the true nature of the so-called persecution. It would hardly do for them to confess that all the persecution they get consists in the efforts of boards of health and medical societies to compel them to be honest and tell the public the plain truth about themselves, or at enforcing laws against dangerous ignorance.—*American Medical Surgical Bulletin.*

